# Hepatoprotective Herbs, Avicenna Viewpoint

**DOI:** 10.5812/ircmj.12313

**Published:** 2014-01-05

**Authors:** Hamid Shamsi-Baghbanan, Afsaneh Sharifian, Somayeh Esmaeili, Bagher Minaei

**Affiliations:** 1 School of Traditional Medicine, Shahid Beheshti University of Medical Sciences, Tehran, IR Iran; 2 Basic and Molecular Epidemiology of Gastrointestinal Disorders Research Center, Shahid Beheshti University of Medical Sciences, Tehran, IR Iran; 3 Traditional Medicine and Materia Medica Research Center, School of Traditional Medicine, Shahid Beheshti University of Medical Sciences, Tehran, IR Iran; 4 Department of Traditional Medicine, Tehran University of Medical Sciences, Tehran, IR Iran

**Keywords:** Plants, Hepatoprotective, Avicenna, Medicine, Traditional, Liver, Complementary Therapies

## Abstract

**Background:**

Liver injury or dysfunction is considered as a serious health problem. The available synthetic drugs to treat liver disorders are expensive and cause further damage. Hence, hepatoprotective effects of some herbal drugs have been investigated, and one of the methods to choose herbs in order to study their biological effects is to search in ancient medical texts. Avicenna who is known as the prince of physicians had collected and classified Greek, Persian and Islamic medicine in the best possible way in the book of Canon in Arabic.

**Objectives:**

Avicenna’s book of The Canon of Medicine was reviewed to find the hepatoprotective herbs.

**Patients and Methods:**

Three different versions of the Canon were prepared and utilized. To find scientific names of plants we took advantage of three botany references. All of the herbs were investigated on the basis of scientific data from hepatoprotective effects point of view. The searched term was “hepatoprotective” without narrowing and limiting. The searched databases included Cochrane library, Web of science, SID, Irandoc and IranMedex.

**Results:**

18 plants were found. 85% of the presented species, genus or families of plants were reported to have hepatoprotective properties and in the remaining 15% there were no reports of hepatoprotective effect. Flowers and fruits were the most used part of the plants. Most of the plants had simultaneous protective effects on multiple organs but the protective effect on the liver was mostly accompanied by protective effect on the stomach (83%). The average temperament of these herbs is "hot" in the 2nd phase of the 2nd grade, and "dry" in the 3rd phase of the 2nd grade. Hepatoprotective herbs mostly prescribed as a part of hepatoprotective compound drugs formula or other formula for liver diseases are *Crocus sativus*, *Pistacia lentiscus*, and *Cinnamomum spp*.

**Conclusions:**

Maybe there is common mechanism for protecting both liver and stomach. *Aquilaria agallocha*, *Aquilaria malaccensis*, and *Ruscus aculeatus* whose hepatoprotective effects have not yet been reported are considered as good candidates for future investigations. Given that *Crocus sativus*, and *Cinnamomum spp* are used as flavors in most countries, they will be introduced for more investigation in order to produce hepatoprotective drugs.

## 1. Background

Liver as the largest internal organ in the body, plays a crucial role in many essential physiologic processes and is vulnerable to a wide variety of toxic, microbial, metabolic, circulatory, and neoplastic insults. Surveillance studies in the United States document an annual incidence of newly diagnosed chronic liver disease of 72 per 100,000 populations (1) and Liver diseases are considered as one of the most serious health problems. On the other hand, treatment options for common liver diseases are limited, and therapy with modern medicine may lack efficacy. The effectiveness of treatments such as those using corticosteroids and interferon is consistent, carries the risk of adverse effect, and is often too costly (2). Hence, we are in the need of new drugs with minor side effects. Clinical studies demonstrated efficacy and safety of a number of herbal products in the treatment of liver diseases (3). Amongst the most important and proven cases of using these herbs is utilizing them as hepatoprotective agents (4). Studies revealed the value and worthiness of investigating Greek, Latin and other medical scripts in order to get familiar with herbs, and choose them for pharmacological studies (5).

Avicenna, one of the most famous physicians of the old era who is known as "the prince of physicians" in the west is the author of The Canon of Medicine. The Canon of Medicine presents a clear and organized summary of all the medical knowledge of the time including Greek, Persian and Islamic medicine. The Canon is considered as one of the most famous books in the history of medicine (6). On the other hand, evidence indicates that detailed study of the Canon and comparing it with new findings can lead to finding plants having biological effects. Hence, in this research we investigated the Canon to find herbs which have hepatoprotective effect from the viewpoint of Avicenna.

The Canon of Medicine is composed of five volumes. The first book is about general Anatomy and principles of medicine, and the second book deals with Materia Medica. The third book covers the function and diseases of each organ. Book 4 is about diseases that affect the whole body like fevers, and book 5 deals with compound drugs. In the 2nd book, about 800 materia were introduced which are mostly herbal (6). In this book, each medicinal property was described with specific terms. For example, Moghavi is an effect defined as follows: A drug effect which moderates the disposition and temperament of an organ to an extent so that it prevents the superfluous matter and disorders moving toward it (7).

A drug effect with a Moghavi property prevents liver from injuries or diseases. This definition is similar to hepatoprotective effect. Hepatoprotective agents are those compounds, which mitigate the liver injury caused by hepatotoxic agents (8) thus can prevent damage to the liver. Although some scholars considered fortifying, tonic and strengthening (7).

## 2. Objectives

 Considering the aforementioned definition, and as the English equivalent for Moghavi, protective is a more suitable and accurate equivalent. Therefore, it seems that a drug with Moghavi effects on liver is a hepatoprotective drug, therefore the Canon was investigated to find herbs having such properties.

## 3. Materials and Methods

**Figure 1.**
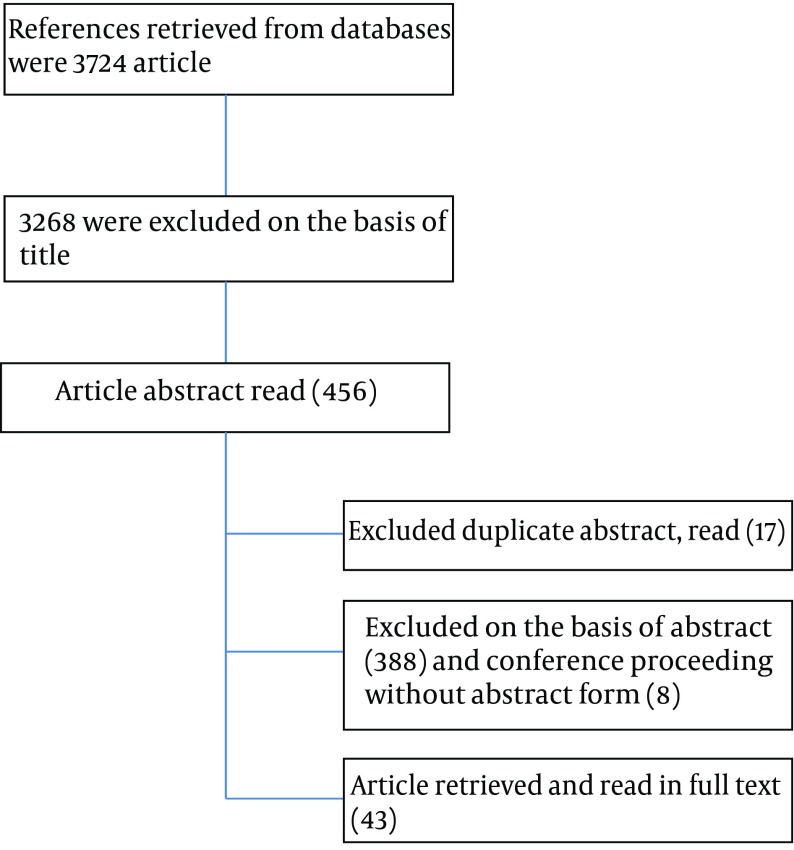
Flow of the Study

Three different versions of the Canon were prepared and utilized: 1- the corrected version of Canon in Arabic (9), 2- Arabic manuscript of the Canon (10), and 3- Translated version of the Canon in English (7). As the first step, to indicate which hepatoprotective herbs or compound drugs Avicenna had prescribed for liver diseases, the 3rd volume of the Canon under Liver Disease topic were searched. To find the scientific names of plants we took advantage of three botany references (11-13) and if two or three books agreed about that name, the scientific name was chosen; if the scientific names of plants were not found, the botanical descriptions were recorded instead. Eventually, the reports of their hepatoprotective effect in the available articles were investigated. And then the term “hepatoprotective” was searched without narrowing and limiting search elements only in the English articles. Liver protective drugs were also extracted. The searched databases included Cochrane library, Web of science, SID, Irandoc and IranMedex up to June 8, 2013. All human and animal studies that included the evidences of the effects of hepatoprotective herbs with any outcomes were selected for the review. Clinical trials (any phase) were identified for data abstraction and observational studies. Only publications without available abstracts and letters to the editor were excluded from the review. Unpublished data were also excluded from the study. Duplication was avoided by excluding review of multiple copies of the same article in several databases. Flow of the study is in Figure 1. 

## 4. Results

Avicenna introduced 19 herbal parts as hepatoprotective that their characteristics are given in Table 1. Old names of hepatoprotective compound drugs, plants used in them, their usage and frequency of their prescription in liver disease which is mentioned in the 3rd volume of the Canon are given in Table 2. Since the components of Marham Dawa Al-Amdhun as one of the hepatoprotective compound drugs was not found in the Canon and other available pharmacology books, this drug was not mentioned in the Table 2. 

**Table 1.** Data from the Second Volume of the Canon about Hepatoprotective Herbs

Traditional name	Family	Scientific Name	Plants Temperament	Organs Which the Herb has Protective Effect on	Used Part	Frequency of Use in Liver Disease		Pharmacological Model Used to Examine Hepatoprotective Effect
						As a Part of Hepatoprotective Compound Drugs	As a Part of Other Compound Drugs	
**Ambarbaris**	Berberidaceae	Berberis vulgaris (11-13)	"cold" in the third phase of the third grade, "dry" in the third phase of the third grade	Liver, stomach	Fruit	0	10	Mice/CCl4 (14)
**Joze boa**	Myristicaceae	Myristica fragrans (11-13)	"hot" in the last phase of second grade, Dry in the last phase of second grade	Liver, stomach, spleen, eye	Kernel of seed	1	1	Cisplatin/ Mice (15)
**Basbaseh**	Myristicaceae	Myristica fragrans (11-13)	"hot" in the second grade, "dry" in the second grade	Liver, stomach	Peel of seed	0	1	Mice/Cisplatin (15)
**Darchini**	Lauraceae	Cinnamomum zeylanicum (11, 12), C. cassia (12, 13), C. verum (11, 13)	"hot" in the third grade, "dry" in the third grade	Liver, stomach	Stembark	20	8	Rat/CCl4 (16), Mice/Ethanol (17), Rat/CCl4 (18)
**Zafaran**	Iridaceae	Crocus sativus (11-13)	"hot" in the first grade, "dry" in the first grade	Viscera, stomach, ,heart, lung, liver	Stigma and style	19	28	Mice/Rifampin (19)
**oufd**	Thymelaeaceae	Aquilaria agallocha (11, 13), A. malaccensis (11, 13)	"hot" in the second grade, "dry" in the second grade	Viscera, Heart, Stomach, liver	stem	1	3	- ^a^
**Ghafeth**	Asteraceae, Rosaceae	Eupatorium cannabinum (11, 12), Agrimonia eupatoria (12, 13)	"hot" in the first grade, "dry" in the second grade	liver	flower	0	20	Rat/CCl4 (20), Rat /Ethanol (21)
**Qaranful**	Myrtaceae	Syzygium aromaticum (11-13)	"hot" in the third grade, "dry" in the third grade	Stomach, liver	Dried buds	10	2	Rat/Paracetamol (22)
**Luk**	Terebinthaceae	Extract from this family (11, 13)	"hot" in the second grade, "dry" in the third grade	liver	Gum	1	12	- ^b^
**Murdesfaram**	Asparagaceae	Ruscus aculeatus (11-13)	"hot" in the second grade, "dry" in the second grade	Stomach, liver	Flower and leaf	0	1	- ^a^
**Waj**	Araceae	Acorus calamusl (11-13)	"hot" in the first phase of the second grade, "dry" in the first phase of the second grade	Stomach, cold liver	Root	0	3	Rat/Paracetamol (23)
**Hil boa**	Zingiberaceae	Elettaria cardamomum (11-13)	"hot" in the second grade, "dry" in the second grade	Cold liver, cold stomach	Fruit	1	0	Rat/CCl4 (24)
**Mastaki**	Anacardiaceae	Pistacia lentiscus (11-13)	"hot" in the second grade, "dry" in the second grade	Liver, Stomach, small intestine	Gum	9	34	Rat/CCl4 (25)
**Balsan**	Burseraceae	Commiphora opobalsamum (11-13)	"hot" in the second grade, "dry" in the second grade	liver	Wood, gum, fruit	9	6	Mice/CCl4 (26)
**Usutkhuddus**	Labiatae	Lavandula stoechas (11-13)	"hot" in the first grade, "dry" in the second grade	Viscera, urinary organ, body	Flower	0	1	- ^c^ (27)
**Armak**	-	A Yemenite fragrant rind like Cinnamon	"hot" in the last phase of the second grade, "dry" in the last phase of the second grade	Viscera, heart, brain	-	0	0	
**Ward**	Rosaceae	Rosa damascene (11-13)	Cold in the first grade, "dry" in the first phase of the second grade	Internal organs	Petals	0	22	Rat/CCl4 (28)
**Kashuth**	Cuscutaceae	Cuscuta monogyna (11-13)	"hot" in the first phase of first grade, "dry" in the last phase of second grade	Stomach, liver	Whole Plant and seed	0	11	- ^d^ (29)
**Sak**	Euphorbiaceae	Phyllanthus emblica (11-13)	"hot" in the first grade, "dry" in the second grade	viscera	fruit	0	1	Rat /Ethanol (30)

^a^ Based on our research, the hepatoprotective effect of this species, genus, and family have not been studied.

^b^ Based on our research, the hepatoprotective effect of this family has not been studied.

^c^ Based on our research, although in this family hepatoprotective effect was reported but this species and genus have not been studied.

^d^ Based on our research, although in this genus hepatoprotective effect was reported the species has not been studied.

**Table 2.** Properties of Hepatoprotective Compound Drugs Extracted From the 5th Volume of the Canon

Traditional Name of Compound Drugs ^a^	Amberbaris	Jauz buwwa	Basbaseh	Darchini	zafaran	oud	Ghafith	Qaranful	Luk	Murdasfaram	Waj	Hil bawwa	Mastaki	Balsan	Usutkhuddus	Armak	Ward	Kashuth	suk	Frequency of citation in liver diseas	Intake method
**A ^b^**	-	-	-	√	-	-	-	-	-	-	√	√	-	-	-	-	-	-	-	0	oral
**malumali**	-	-	-	-	√	-	-	-	-	-	-	-	-	-	-	-	-	-	-	0	oral
**al-habb al-jami**,** made by Ibn Jahan**	-	-	-	√	√	-	√	-	-	-	-	-	√	√	-	-	√	-	-	0	oral
**Dawa-al-lak al-akbar**	-	-	-	√	√	-	-	√	-	-	-	-	√	√	-	-	-	-	-	7	oral
**Dawa-al-lak al-asghar**	-	-	-	-	-	-	-	-	√	-	-	-	-	-	-	-	-	-	-	0	oral
**habb –e-Astmehighon attributed to al-kindi**	-	-	-	√	√	-	-	√	-	-	-	-	√	√	-	-	-	-	-	1	oral
**Marham –be- shahm-e- hanzal**	-	-	-	-	-	-	-	-	-	-	-	-	-	-	-	-	-	-	-	1	Topical
**Dawa –al- kurkum**	-	-	-	√	√	-	-	-	-	-	-	-	-	-	-	-	-	-	-	10	oral
**Jawarish-e- khuzi**	-	√	√	-	-	-	-	√	-	-	-	-	-	√	-	-	-	-	-	0	oral
**Jawarishn-e- jalinus**	-	-	-	√	√	√	-	√	-	-	-	√	√	√	-	-	-	-	-	0	oral
**Majun- al-khabth**	-	√	-	√	-	-	-	√	-	-	-	√	-	√	-	-	-	-	-	1	oral
**Jawarishn -al-darsini**	-	-	-	√	-	√	-	√	-	-	-	-	-	-	-	-	-	-	-	0	oral
**Safuf-e-ebadat**	-	-	-	-	-	-	-	-	√	-	-	-	√	-	-	-	-	-	-	0	oral
**B ** ^**b**^	-	√	√	-	√	-	-	√	-	-	-	√	√	-	-	-	-	-	-	0	oral
**Tiryaq-e- kabir**	-	-	-	√	√	√	-	-	-	-	√	-	-	√	-	-	-	-	-	0	oral
**Majun-al-kinidi**	-	-	-	-	√	-	√	-	-	-	-	-	-	√	-	-	-	-	-	0	oral
**Majun-al-mosk**	-	-	-	√	√	√	-	√	√	-	-	-	√	-	-	-	-	-	-	1	oral
**Majun-e- shajarina-e-kabir**	-	-	-	√	√	-	-	-	-	-	-	-	-	-	-	-	-	-	-	0	oral
**Majun-e- shajarina-e-sagir**	-	-	-	√	√	-	-	-	-	-	-	-	-	-	-	-	-	-	-	0	oral
**Majun-e-anqardia**	-	-	-	-	√	√	-	√	-	-	-	-	√	-	-	-	-	-	-	0	oral
**Total sum**	0	3	2	12	13	5	2	9	3	0	2	4	8	8	0	0	1	0	0		

^a^ Other plants were also used in formulation of these drugs but only hepatoprotective herbs were mentioned.

^b^ There is no Specific Name for it in the Canon.

The found plants belong to 17 different families. Seventy-five percent of the plants found in this research are reported to have confirmed hepatoprotective effects just the same way as their scientific names. Such reports are also about genus of 5% of plants and family of 5% of plants. There is no report about hepatoprotective effect of 15% of plants composed of 3 plants. The parts of plants used are as follows:

Flower or flower parts: 6 items (27.27%); fruit: 4 items (18.18%); grain or parts of grain: 3 items (13.63%); Stalk or stem bark: 3 items (13.36%); plants gum: 3 items (13.63%); leaf: 1 item (4.54%); root: 1 item (4.54%); the whole plant: 1 item (4.54%). With regard to Table 1, the most common hepatoprotective plants prescribed for liver disease under the formulation of hepatoprotective compound drugs are respectively as follows: *Cinnamomum spp* (Darchin), *Crocus sativus* (Zafaran), *Syzygium aromaticum* (Qaranful), *Pistacia lentiscus* (Mastaki), and *Commiphora opobalsamum* (Balsan). Taking Table 1 into account, most of the hepatoprotective herbs prescribed in liver disease, in formulations other than hepatoprotective compound drugs, are respectively as follows: Mastaki, Zafaran, Ward, Ghafeth, Luk, Kashuth, Ambarbaris, and Darchin. Considering Table 2, the plants frequently used in formulation of compound drugs are respectively as: Zafaran, Darchin, Qaranful, Balsan, and Mastaki.

## 5. Discussion

The word "Moghavi", and its synonyms, which means "Protective" and also words such as "Jeghar" and "Kabed" which both mean "Liver" were searched in the 2nd volume of the Canon. Every time one of these words was found, the due text was studied to determine which plant it is about, and the relation with its hepatoprotective effect was investigated. Traditional medicine of Iran is based on the theory of the presence of Nature in human organs, and that each organ and part of the body is eligible to have a specific amount of "cold" or "hot" nature and also a certain amount of "moist" or "dry" nature. An effect of drugs on body is variation of quality of Natures. If a drug changes the temperament of body toward Hot, it is said to have "hot" temperament and if it changes the nature of body to "cold" it is called to have "cold" temperament, and so forth for other natures. Ability of drugs to change the nature is divided into four grades and each grade is composed of 3 phases. The higher the grade of a drug, the more its ability to change body nature , hence a drug with the 1st grade will slightly change the nature of organ but causes no change in its functionality. The 2nd grade drug makes minor changes in nature and functionality but does not harm the organ. A drug of the 3rd grade alters nature and functionality of organ considerably and may lead to organ damage, but does not cause its death. Drugs in their 4th grade are strong enough to lead to death of organ. It is noteworthy that drug effectiveness in phase 3 of the same grade is much more than equivalent drug in phase 2 of the same grade and effectiveness of phase 2 of the same grade would be more than effectiveness of phase 1 for that grade.

Temperament of 89% of plants is "hot" and "dry". This may indicate that in order to have suitable hepatoprotective effect, temperament must be "hot" and "dry". The average temperament of these plants was "hot" in the 2nd phase of the 2nd grade and "dry" in the 3rd phase of the 2nd grade. Based on definition of plants` temperament grades and their effects on the body, we can claim that most of the drugs selected on the basis of traditional medicines are safe and relatively with no side effects. Based on our survey in the Canon, 19 herbal parts with hepatoprotective effects were found. Since both joze boa and Basbaseh belong to the same plant and no scientific name was found for Armak, and on the other hand, there were two or three different scientific names for some old plants, 22 scientific names were assigned for evidenced based studies of the 17 recognized plants.

Considering that the plants belong to 17 different families perhaps indicates that the hepatoprotective properties are not unique to a family. Given that the most frequently used parts of plants were flowers or flower parts (27.27%) and fruits (18.18%) implies that these parts of plants may be a better choice for extracting hepatoprotective materials. Most plants have simultaneous protective effect on several organs but the protective effect on liver usually accompanies with protective effect on stomach (83%). This perhaps explains the existence of a joint mechanism for protecting the liver and stomach.

All plants introduced by Avicenna were prescribed as a part of hepatoprotective compound drugs, or other formulas in liver diseases. The present studies have reported the hepatoprotective effect in 85% of these plants; therefore continuation of these studies to make an effective drug for liver diseases is promising. On the other hand, plants such as *Aquilaria agallocha*, *Aquilaria malaccensis*, and *Ruscus aculeatus* whose hepatoprotective effects have not yet been reported are considered as good candidates for future investigations. In a survey (31), hepatoprotective effect of a Unani compound drug with the title of Majoon-e-Dabeed-ul-Ward was proven. Fifty-five percent of plant ingredients used in formulation of this drug is the same as plants introduced in this paper that confirms the right choices of Avicenna.

Ninety percent of the compound drugs are oral intake and the remaining 10% must be rubbed on the body. It is interesting to note that there is no hepatoprotective herb in formulation of drugs which must be rubbed but all of them exist in formulation of oral intake drugs and this indicates that oral prescription is probably a better choice for hepatoprotective effectiveness and verifies that the mechanisms of drug effectiveness in both cases of oral intake and rubbing is different. Considering the fact that often several hepatoprotective herbs have been simultaneously used in formulation of hepatoprotective compound drugs they are likely to have synergistic effects that need to be studied further. Most frequently the prescribed hepatoprotective compound drugs for liver diseases are Dawa-al- kurkum and Dawa-al-lak al-akbar.

Interestingly, both plants of Zafaran and Darchin exist in both formulas and Balsan, Mastaki, and Qaranful are used in Dawa al-lak al-akbar formula and these 5 plants are the ones most frequently used in formulation of compound drugs. The survey results revealed that three most frequently used plants for liver disease are Zafaran (47 cases), Mastaki (43 cases), and Darchin (28 cases), respectively. However, Zafaran and Darchin are two plants that have been mostly used to produce compound drugs. On the other hand, these two plants are being used as the flavor in many countries including Iran. Therefore, these two plants are proposed for more investigation and construction of hepatoprotective drugs.
